# Environmental Variables Explain Genetic Structure in a Beetle-Associated Nematode

**DOI:** 10.1371/journal.pone.0087317

**Published:** 2014-01-31

**Authors:** Angela McGaughran, Katy Morgan, Ralf J. Sommer

**Affiliations:** Department for Evolutionary Biology, Max Planck Institute for Developmental Biology, Tübingen, Germany; Tuscia University, Italy

## Abstract

The distribution of a species is a complex expression of its ecological and evolutionary history and integrating population genetic, environmental, and ecological data can provide new insights into the effects of the environment on the population structure of species. Previous work demonstrated strong patterns of genetic differentiation in natural populations of the hermaphroditic nematode *Pristionchus pacificus* in its La Réunion Island habitat, but gave no clear understanding of the role of the environment in structuring this variation. Here, we present what is to our knowledge the first study to statistically evaluate the role of the environment in shaping the structure and distribution of nematode populations. We test the hypothesis that genetic structure in *P. pacificus* is influenced by environmental variables, by combining population genetic analyses of microsatellite data from 18 populations and 370 strains, with multivariate statistics on environmental data, and species distribution modelling. We assess and quantify the relative importance of environmental factors (geographic distance, altitude, temperature, precipitation, and beetle host) on genetic variation among populations. Despite the fact that geographic populations of *P. pacificus* comprise vast genetic diversity sourced from multiple ancestral lineages, we find strong evidence for local associations between environment and genetic variation. Further, we show that significantly more genetic variation in *P. pacificus* populations is explained by environmental variation than by geographic distances. This supports a strong role for environmental heterogeneity vs. genetic drift in the divergence of populations, which we suggest may be influenced by adaptive forces.

## Introduction

All organisms live in environments that vary through time and space, and the influence of environmental heterogeneity on spatial scales of population divergence and genetic isolation is a fundamental question in evolutionary biology [1]–[4]. Environmentally-driven selection can be responsible for differences among populations, through the imposition of highly variable selection pressures among geographic regions that differ climatically. For example, recent studies in plants [5], fish [6]–[7], and crustaceans [8], have inferred regional scales of local adaptation, driven by distinct environmental conditions among populations. More generally, the spatial distributions of plants and animals are often closely tied to environmental conditions [9], as climatic gradients interact with the physiology of organisms to define their ecological niche. The influence of climate on organism fitness and its potential consequences in determining niche structure among populations emphasises the importance of understanding the environment as a proximate cause of patterns in species dynamics [10]–[11].

Although much progress has been made in understanding causes and consequences of local and regional changes in genetic diversity of natural populations [5], [7], [8], [12], it remains arduous to interpret patterns of genetic diversity and identify processes responsible for population genetic structure in the wild. This is largely due to the fact that similar genetic patterns may result from processes as diverse as adaptation, dispersal limitation and genetic drift. In addition, processes driving population genetic structure interact at the level of the landscape, rather than acting in isolation [8]. Such patterns are a general focus of population genetics, and evaluating the geographic distribution of genetic diversity based on environmental attributes is a powerful approach for understanding evolutionary processes [7], [12]–[16].

**Figure 1:**
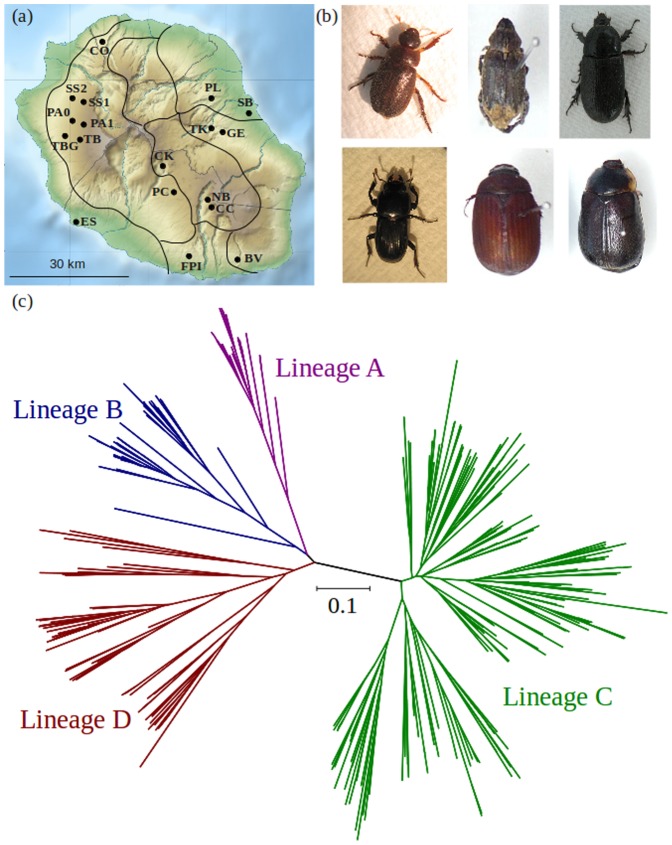
*Pristionchus pacificus* diversity and beetle hosts. (a) Map of La Réunion Island showing the approximate geographic locations (black circles), ecozones (solid lines) and altitudes (visual topography) which were used to examine the effects of environmental variables on genetic structure in *P. pacificus* (see Table 2 and text for further details); (b) Selected photographs of the beetle hosts of *P. pacificus* (from left to right, top row: *Adoretus* sp., *Hoplia retusa, Oryctes borbonicus*; bottom row: *Amneides godefroyi, Maladera affinis, Hoplochelus marginalis*), photographs, taken by Mark Leaver, are not to scale; (c) Neighbour-joining tree created in Phylip ver. 3.69 using 19 microsatellite markers and 370 *P. pacificus* strains, main clusters are colour-coded according to the four described genetic lineages.

Island systems often represent diversity hotspots characterised by high species richness and endemism, resulting from local diversification of geographically or ecologically isolated populations following colonisation [17]. As such, islands are ideal settings for research focused on population genetics. La Réunion Island (2–3 Ma; 2512 km^2^), a component of the Mascarene island chain lying east of Madagascar in the Indian Ocean, is one such biodiversity hotspot [18], offering remarkable opportunities to determine the role of various environmental variables in defining population structure. La Réunion Island is a topographically and ecologically complex volcanically-active island. Inland steep relief, coastal lowlands, volcanic plateaus (>2000 m a.s.l.), and deep valleys demarcate the island into a suite of ecozones, in which species diversity and endemism broadly differ [19]–[20] (Fig. 1a). As such, evolutionary divergence within populations precipitated by environmental differences among regions is likely. Indeed, Delatte et al. [21] found east/west differences in mosquito population structure on Réunion Island that corresponded significantly with the prevalence of environmental conditions. Namely, more suitable larval habitats were present in the wetter eastern parts of Réunion, and gene flow among mosquito populations was much higher in the west of the island where individuals were more likely to migrate in search of suitable habitat sites [21].

*Pristionchus pacificus* is a hermaphroditic nematode of cosmopolitan distribution that forms a non-invasive host-relationship with scarab beetle and other insect taxa [22]–[23]. Its life cycle consists of embryogenesis in four juvenile stages, called J1–J4, and an adult stage. Animals proceed through this typical or direct developmental pattern under favourable food conditions. In contrast, with unfavourable conditions such as the absence of food, high temperature, or high population density, worms enter an arrested dauer stage. Dauer larvae are non-feeding but motile, representing an alternative J3 juvenile stage which can survive for months, resuming development once conditions improve [24]. It is this dauer stage that is responsible for navigating through soil to find and attach to a beetle host. *P. pacificus* is widely distributed across La Réunion Island where it inhabits a broad range of environments, encompassing gradients of altitude, ecozone, geography and beetle host (Fig. 1a,b). As such, this species is an ideal organism for studying the effects of environmental factors on genetic structure.

After an initial description of the distribution of *P. pacificus* on La Réunion [25], detailed population genetic studies that included demographic, clustering, divergence and colonisation analyses of microsatellite (STR) and mitochondrial data were completed [26]–[27]. Both empirical evidence and modelling approaches supported the presence of at least four genetic lineages of *P. pacificus* on La Réunion (Lineages ‘A’, ‘B’, ‘C’ and ‘D’; Fig. 1c) [25]–[27]. These lineages diversified before establishment on the island over multiple independent colonisation events that occurred at different times, from different source locations and in association with different host beetles [26]–[27]. Complex interactions between geography, beetle host and genetic lineage have subsequently resulted in substantial genetic diversity among geographic populations that have been contributed to by multiple genetic sources [26]. However, despite historical mixing, modern populations are significantly differentiated and characterised by low gene flow [26]–[27]. This suggests that local environmental factors may be mediating genetic differences among populations through adaptive processes.

Preliminary findings indicate a genetic delineation between drier western and wetter eastern areas of the island [26] however, research to date has not explicitly tested for a relationship between genetic structure and environmental differences in *P. pacificus*. Here, we present what is to our knowledge the first study to statistically evaluate the role of the environment in shaping the structure and distribution of nematode populations. We exploit the fact that geographically and ecologically isolated hermaphroditic *P. pacificus* populations may respond differently to environmental factors due to local adaptation, despite constraints associated with the historical diversification of lineages. We examine the effect of environmental factors on genetic structure by more densely sampling *P. pacificus* from La Réunion Island, adding 176 new strains to the existing STR dataset [26], and targeting a diverse array of habitats across 18 widespread geographic locations. Based on our complete collections, we use species distribution modelling to define the ecological niche of both *P. pacificus* and its beetle hosts. Next, we perform population genetic analyses to examine the effect of environmental factors on the partitioning of genetic structure. Finally, we combine genetic data with site-averaged multivariate environmental data to explicitly test the hypothesis that genetic structure in *P. pacificus* is influenced by environmental variables, assessing the relative importance of these environmental factors (geographic distance, temperature, precipitation, altitude, and beetle host) on genetic variation.

## Materials and Methods

### Species Distribution Models (SDMs)

We first aimed to determine the ecological niche of *P. pacificus* on La Réunion Island. Species distribution modelling programs identify sites with similar environments to those where a species has already been observed as potential occurrence areas.

#### Species presence data

**Figure 2:**
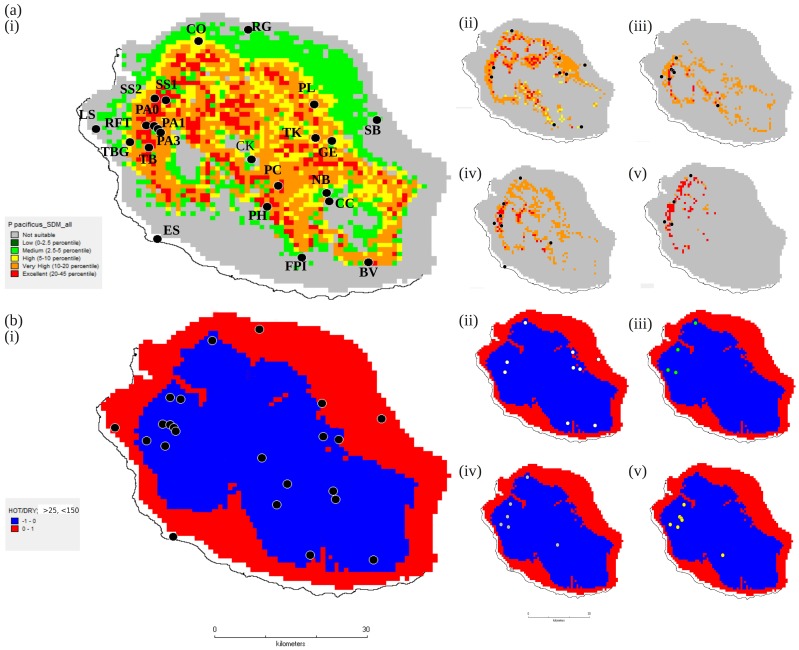
SDMs of *Pristionchus pacificus* and its beetle hosts using DIVA-GIS. (a) Species distribution models for: (i) *P. pacificus,* and (ii-v) its beetle hosts: (ii) *Adoretus* sp., (iii) *Hoplochelus marginalis,* (iv) *Hoplia retusa,* and (v) *Oryctes borbonicus,* on La Réunion Island, draped over a topographical surface at 2.5 arc minute resolution, using the program DIVA-GIS and all bioclimatic variables (see Table 1). Panel (a) demonstrates the geographic distribution of theoretical suitable habitat, with the legend indicating presence probabilities for *P. pacificus* over five levels in a linear series; level 1 represents the minimum probability value of *P. pacificus* presence and level 5, the maximum probability presence value, according to a ‘percentile envelope’ based on the various climate variables in the model (for example, category 1 =  low (0–2.5 percentile) indicates a low presence probability of *P. pacificus*, where dark green areas on the map are those corresponding to climate envelope outliers (in the <2.5 and >97.5 percentile). Species distribution points are indicated with black circles in each case, and Panel (a)(i) shows location codes for all maps. (b) Climatic patterns for La Réunion Island draped over a topographical surface at 2.5 arc minute resolution using the program DIVA-GIS, illustrating the hottest (maximum temperature of warmest month >25°C) and driest (precipitation of driest quarter <150 mm) areas (in red). Panels show individual species distributions (coloured circles) for: (i) *P. pacificus,* and (ii-v) its beetle hosts: (ii) *Adoretus* sp., (iii) *H. marginalis,* (iv) *H. retusa,* and (v) *O. borbonicus*. Panel (a)(i) shows the location codes for all maps.

Species occurrence data corresponded to 23 geographic Réunion locations from which *P. pacificus* had been successfully sampled on an annual basis between 2008 and 2012 (Fig. 2a). In all cases, nematode sampling on the island involved collection of beetles at light traps, and collection at all locations was authorised by Parc National de La Réunion (no ethics statement was required). Beetles were then sacrificed onto nutrient agar plates and monitored daily for the emergence of nematodes. Single emerging adult nematodes were picked onto new plates and maintained with OP50. Upon return to Germany, isogenic lines were created from all single adult *P. pacificus* hermaphrodites by allowing them to reproduce and maintaining their offspring in culture. These lines were maintained in the laboratory for at least 10 generations and then frozen to form part of the Sommer laboratory nematode strain database.

#### Environmental data

Climate data for La Réunion Island was downloaded from the WorldClim website (ver. 1.3, October 2004; http://www.worldclim.org/). These data encompass biologically relevant temperature and precipitation layers created by interpolating observed climate from climate stations around the world using a thin-plate smoothing spline set to a resolution of approximately 1 km (2.5 arc minutes), over the 50-year period from 1950 to 2000 [28]. As well as maximum, minimum and mean monthly temperature and precipitation estimates, 19 derived bioclimatic variables were downloaded. These 19 parameters incorporate annual trends (e.g. mean annual temperature, annual precipitation), aspects of seasonality (e.g. annual range in temperature and precipitation) and extreme or potentially limiting environmental factors (e.g. temperature of the coldest and warmest months, and precipitation of the wettest and driest months) (Table 1).

**Table 1:** Bioclimatic variables.

Code	Environmental variable
BIO1	Annual mean temperature
BIO2	Mean diurnal range (Mean of monthly (maximum temperature - minimum temperature))
BIO3	Isothermality (BIO2/BIO7)(*100)
BIO4	Temperature seasonality (Standard deviation of temperature * 100)
BIO5	Maximum temperature of warmest month
BIO6	Minimum temperature of coldest month
BIO7	Temperature annual range (BIO5– BIO6)
BIO8	Mean temperature of wettest quarter
BIO9	Mean temperature of driest quarter
BIO10	Mean temperature of warmest quarter
BIO11	Mean temperature of coldest quarter
BIO12	Annual precipitation
BIO13	Precipitation of wettest month
BIO14	Precipitation of driest month
BIO15	Precipitation seasonality (Coefficient of variation)
BIO16	Precipitation of wettest quarter
BIO17	Precipitation of driest quarter
BIO18	Precipitation of warmest quarter
BIO19	Precipitation of coldest quarter

Bioclimatic variables used for *Pristionchus pacificus s*pecies distribution models in the programs DIVA-GIS and MAXENT. Variables (sourced from: Worldclim ver. 1.3, October 2004; http://www.worldclim.org/) are derived from tmean, tmin, tmax and prec (average monthly mean, minimum and maximum temperature, and average monthly precipitation, respectively).

#### SDM analysis

To model the habitat of *P. pacificus*, two SDM-based techniques were employed. First, DIVA-GIS ver. 7.5 [29] was used for mapping and geographic data analysis. As input, DIVA-GIS requires standard map data files such as ESRI shapefiles, climate data, and species occurrence data. Réunion Island administrative areas shapefiles were downloaded from http://www.diva-gis.org/gData and imported alongside species occurrence and climate data into DIVA-GIS, where the Bioclim option was used to model species distribution. Because inherent multicollinearity among climatic variables often makes it difficult to distinguish among them and their effects on given response variables [30]–[32], while inclusion of large numbers of climate variables in such models may lead to over-parameterisation [33], three subsets of climate data were used in the analysis. These included: (1) all bioclimatic variables; (2) ‘summary; climate variables (BIO1–BIO4, BIO7–BIO12, BIO15; Table 1), including annual, range and mean variables; and (3) only ‘extreme’ climate variables (BIO5, BIO6, BIO13, BIO14; Table 1), including maximum and minimum, wettest and driest variables. All predictions used the maximum extent option in DIVA-GIS, resulting in continuous predictions of the potential distribution of *P. pacificus* on La Réunion Island.

The program MAXENT ver. 3.3.3 [34]–[35] was also used for species habitat modelling to confirm agreement among different SDM methods. MAXENT employs a maximum-entropy approach, taking as input layers of environmental values and geo-referenced species occurrence data, and distinguishing species presence from random. Like DIVA-GIS, MAXENT produces a model of the range of the given species. As for DIVA-GIS, different subsets of climate data were used in each analysis, and in MAXENT runs, 25% (n = 5) of sample records were used for testing, which enabled the program to calculate a testing vs. training data comparison. Specifically, an AUC (Area Under the Curve) value was generated, and this provided a measure of the predictive power of a given model – Araújo et al. [36] suggested that AUC values should exceed 0.70 in order to provide acceptable predictive power. In MAXENT runs, the logistic output format was used and a 10 percentile training presence was enforced (this reflects the probability value at which 90% of the presence points fall within the potential area; the remaining 10%, which fall outside the potential area, are those with an atypical environment, and are not included within the limits of the realized niche). While the MAXENT model was being trained, the environmental variables that were making the greatest contribution to the model were recorded. In this way, MAXENT runs could be used to determine which climatic variables may be most important in determining species distributions. This was further explored using the ‘jackknife’ option in MAXENT, whereby a model is created using each variable in isolation for comparison to a model created using all variables. For all runs performed in MAXENT, 10 replicates were performed for cross-validation.

In addition to generating models for *P. pacificus*, SDMs were generated under the same settings described above in both DIVA-GIS and MAXENT for the four beetle hosts of *P. pacificus* for which we had >3 collection locations (see Table 2,3). Combinations of climate variables were also applied to maps in conjunction with species presence points (both *P. pacificus* and its beetle hosts) to generate visualisations of the association between species distribution and the hottest/driest or coldest/wettest areas of the island.

**Table 2:** Sampling codes and classification schemes.

Population	Population code	Altitude	Ecozone	Beetle-host(s)	Genetic cluster(s)	Genetic lineage
Base Vallée	BV	2	3	Ado	2,5,6,12	C,D
Le Cratère Commerson	CC	5	5	Amn	3,10	B
Coteau Kerveguen	CK	5	5	Amn	10	B
Colorado	CO	2	1	Ado,Hch,Hop	1,6,7,9,11	C
Etang Salé	ES	1	6	Hop,Mal	6,11	C
Forêt Petite Ile	FPI	3	4	Ado	9	C
Grand Etang	GE	2	3	Ado,Chr,Soil	1,5,6,7,8,9,11,12	A,C,D
Nez de Bœuf	NB	5	5	Amn,Soil	10	B
Des Palmistes_0	PA0	3	1	Hop	1,4	A,C
Des Palmistes_1	PA1	4	1	Ado,Ory	7,10	B,C
Plaines des Cafrès	PC	4	1	Hop	6,9,11	C
Plaines des Lianes	PL	2	2	Ado	5,7,12	C,D
Saint Benoit	SB	1	2	Ado,Aph,Mal	4,6,11	A,C
San Souci_1	SS1	4	1	Ory	2,6,9,11,12	C,D
San Souci_2	SS2	3	1	Hch,Hop	2,7	C
Trois Bassin	TB	4	1	Ado,Hch,Hop,Ory	1,2,5,6,7,8,9,11	C,D
Trois Bassin Garden	TBG	3	1	Aph,Ata,Hch,Hop,Mal,Ory	1,6,9,11	C
Takamaka	TK	2	3	Ado	5	D

Sampling codes for 18 *Pristionchus pacificus* populations on La Réunion Island, and their classification by altitude, ecozone, beetle host, genetic cluster and genetic lineage. Although several significant altitude combinations were tested, final altitude group designations correspond to: (1) <100 m; (2) 500–800 m; (3) 900–1,200 m; (4) 1,300–1,600 m; (5) 2,000–2,300 m; Beetle host codes correspond to: Ado: *Adoretus* sp.; Am: *Amneidus godefroyi*; Aph: *Aphodius* sp.; Ata: *Ataenius* sp.; Chr: *Chrysomelidae* sp.; Hch: *Hoplochelus marginalis*; Hop: *Hoplia retusa*; Mal: *Maladera affinis*; Ory: *Oryctes borbonicus*; Genetic cluster designation based on the 12 genetic clusters identified using STR data in the program INSTRUCT. See also Figure 1.

**Table 3:** INSTRUCT results.

Cluster number	Proportion of individuals in each cluster (S.E.M.)
1	0.069(0.012)
2	0.014(0.005)
3	0.050(0.009)
4	0.084(0.012)
5	0.082(0.013)
6	0.068(0.012)
7	0.049(0.010)
8	0.096(0.013)
9	0.126(0.015)
10	0.122(0.016)
11	0.144(0.015)
12	0.094(0.012)

Mean+S.E.M. of population assignments to each cluster detected by INSTRUCT for 370 individuals of *Pristionchus pacificus* on La Réunion Island.

### Population Genetic Structure

SDMs allowed us to examine how environmental factors may be governing the presence of *P. pacificus* in La Réunion habitats. Next, we aimed to examine genetic structure of *P. pacificus* and assess the effects of environmental factors on this structure. From the Sommer collection, a subset of ∼200 strains had been typed for STR markers in a previous study [26] (available from Dryad, accession number: doi:10.5061/dryad.ns1stov2). An additional 176 strains were genotyped at STR markers for the current study (Dryad accession number: doi:10.5061/dryad.8883h), culminating in a final STR dataset of 370 strains from 18 geographic La Réunion Island locations (Table 2) and eight beetle host species, and consisting of 19 loci that span the genome (six chromosomes) of *P. pacificus*. See [26] for information on development and generation of STR markers.

A genetic clustering program was used to probabilistically assign *P. pacificus* individuals to genetic clusters. Although many programs are currently available for this type of analysis, INSTRUCT ver. 1.0 [37] was chosen as the best model for use here. INSTRUCT is an extension of STRUCTURE [38], designed to accommodate selfing – rather than assuming random mating and Hardy-Weinberg equilibrium (HWE) within populations, INSTRUCT avoids potential bias by allowing selfing or inbreeding rates to vary between clusters. Five independent MCMC chains were run in INSTRUCT, each with 1,000,000 iterations, a burn-in of 500,000 and a thinning interval of 100 between retained draws. Tested values of K ranged from 1–25, and the optimal K-value was selected based on deviance information criteria and using the ΔK method of Evanno et al. [39]. INSTRUCT runs were replicated several times, checked for concordance, combined using CLUMPP ver. 1.1.2 [40], and graphically displayed using DISTRUCT ver. 1.1 [41]. To complement the INSTRUCT analyses, the adegenet [42] package in R ver. 2.1.5.0 [43], was used to perform Principal Component Analysis (PCA) on the STR dataset, identifying the axes of greatest differentiation between populations. In each case, missing data (9.7%) were first replaced using the scaleGen function, which replaces all NA values with the mean allele frequency.

We hypothesised that environmental variables would have significant effects on genetic structure, diversity and differentiation among populations. To test this, we partitioned the STR dataset according to four relevant environmental classifications: (a) geographic sampling locality; (b) ecozone; (c) altitude; (d) host beetle species (Table 2). The ecozone classification used for (b) followed original definitions of Météo-France [20]. These were based on the application of several methods of classification using annual precipitation and annual distribution of dry and rainy days data, with seven rainfall regions finally defined [20]. We used these to determine ecozone classifications for the populations of the current study by matching our geographic locations to the relevant Météo-France regions (Fig. 1a; Table 2,3). Although several significant (using AMOVA tests, see below) altitude combinations were tested, the final altitude classification was chosen to correspond to five groups whereby each location was within ∼100 m altitude of the mean altitude for its category – in this way, similar altitudes were grouped into categories together. Results from the INSTRUCT and PCA analyses (above) were reproduced based on these four partitioned datasets using the same settings as those employed on the non-partitioned dataset. To explore the effect of unbalanced sample sizes between ecozones, geographic locations and clusters (for which two, four, and one population had a sample size <5, respectively; Table 4), we performed the PCA analysis both with all populations, and with only populations for which n>5. Using ARLEQUIN ver. 3.5.1.2 [44] on both the non-partitioned and partitioned datasets, we also estimated measures of diversity: number of alleles, allelic size range, gene diversity (He) and theta H; analysed the partitioning of molecular variance (AMOVA); and measured population differentiation (pair-wise Rst), with significance of statistics determined over 10,000 permutations.

**Table 4:** Sampling sites and statistics of genetic variation.

	Population code	n	He(s.d.)	T_H_	An(s.d.)	As(s.d.)
(a)	eco1	195	0.580(0.296)	1.553	12.579(9.907)	25.278(24.129)
	eco2	44	0.641(0.153)	1.672	6.053(2.738)	18.684(20.451)
	eco3	77	0.629(0.209)	1.643	9.368(6.011)	23.368(17.363)
	eco4	4	–	–	1.000(0.000)	–
	eco5	47	0.418(0.307)	1.555	4.684(2.868)	10.684(9.322)
	eco6	3	0.418(0.291)	1.556	2.000(0.745)	10.571(17.592)
	Mean	62	0.5372(0.111)	1.596	5.947(4.412)	17.717(6.902)
(b)	BV	10	0.463(0.229)	1.511	2.842(1.068)	13.706(16.680)
	CC	22	0.248(0.226)	2.180	1.895(0.737)	3.615(3.254)
	CK	7	0.319(0.318)	1.800	2.000(1.247)	9.000(8.112)
	CO	34	0.520(0.283)	1.503	4.632(3.218)	14.812(17.619)
	ES	3	0.273(0.300)	2.016	1.474(0.513)	6.778(8.643)
	FPI	4	-	-	1.000(0.000)	-
	GE	63	0.625(0.213)	1.633	8.842(5.560)	21.947(17.690)
	NB	18	0.368(0.296)	1.648	3.053(1.840)	10.333(8.583)
	PA0	3	0.435(0.239)	1.534	1.737(0.733)	7.933(9.130)
	PA1	8	0.326(0.252)	1.775	2.263(1.195)	12.571(20.832)
	PC	24	0.481(0.293)	1.503	3.895(2.536)	13.647(19.006)
	PL	13	0.384(0.272)	1.614	2.947(1.508)	14.312(13.001)
	SB	31	0.535(0.175)	1.510	4.316(1.734)	16.737(20.626)
	SS1	13	0.460(0.283)	1.513	3.368(1.674)	13.176(15.047)
	SS2	7	0.287(0.289)	1.946	1.947(0.970	14.700(16.839)
	TB	80	0.542(0.293)	1.514	8.737(6.797)	21.444(22.807)
	TBG	26	0.502(0.310)	1.500	4.684(2.926)	15.588(16.549)
	TK	4	0.244(0.304)	2.210	1.474(0.905)	12.250(13.177)
	Mean	21	0.412(0.116)	1.701	3.395(2.257)	13.091(4.724)
(c)	alt1	34	0.582(0.155)	1.555	4.895(1.997)	17.789(20.582)
	alt2	124	0.678(0.201)	1.789	11.579(7.876)	25.368(16.866)
	alt3	40	0.562(0.298)	1.531	6.474(3.821)	20.556(23.988)
	alt4	125	0.556(0.293)	1.526	10.474(8.222)	22.889(24.159)
	alt5	47	0.418(0.307)	1.555	4.684(2.868)	10.684(9.322)
	Mean	74	0.559(0.093)	1.591	7.621(3.209)	19.457(5.650)
(d)	ado	126	0.681(0.207)	1.803	12.105(8.055)	26.316(24.855)
	amn	39	0.400(0.296)	1.583	3.947(2.571)	9.833(8.487)
	aph	15	0.575(0.178)	1.547	3.421(1.216)	12.611(12.962)
	ata	8	0.373(0.310)	1.637	2.211(1.134)	11.077(12.003)
	Chr	1	–	–	–	–
	hch	38	0.526(0.313)	1.505	5.632(4.126)	15.667(17.064)
	hop	51	0.575(0.282)	1.546	7.053(4.672)	17.444(18.573)
	mal	18	0.544(0.151)	1.515	3.737(1.695)	14.895(19.950)
	ory	64	0.550(0.286)	1.520	8.526(5.976)	21.556(22.783)
	soi	10	0.527(0.233)	1.506	3.789(1.398)	17.167(21.355)
	Mean	41	0.528(0.093)	1.574	5.602(3.137)	16.285(5.185)
(e)	clu1	33	0.508(0.270)	1.500	5.368(3.287)	16.765(17.960)
	clu2	37	0.396(0.290)	1.591	4.368(2.773)	14.438(16.046)
	clu3	17	0.149(0.214)	3.454	1.579(0.769)	4.000(3.817)
	clu4	26	0.356(0.251)	1.681	3.263(1.408)	7.789(9.157)
	clu5	47	0.456(0.288)	1.516	5.526(3.835)	16.611(12.976)
	clu6	27	0.487(0.334)	1.501	4.737(3.016)	16.118(19.496)
	clu7	21	0.447(0.306)	1.522	3.526(2.458)	16.133(17.246)
	clu8	4	0.635(0.189)	1.657	2.579(0.692)	9.444(7.221)
	clu9	48	0.473(0.324)	1.506	5.368(4.044)	17.235(25.560)
	clu10	31	0.408(0.319)	1.570	4.263(2.806)	11.235(9.737)
	clu11	53	0.468(0.296)	1.508	6.263(4.724)	18.056(21.805)
	clu12	26	0.460(0.260)	1.513	4.105(2.424)	13.167(14.415)
	Mean	31	0.437(0.114)	1.710	4.245(1.344)	13.416(4.413)

Sampling sites and statistics of genetic variation for *Pristionchus pacificus* populations on La Réunion Island at 19 microsatellite loci for STR datasets: (a) ecozone; (b) geography; (c) altitude; (d) beetle host; and (e) genetic cluster. In all cases, n – sample size; He – expected heterozygosity; T_H_ – Theta H; An – number of alleles; As – allelic size range; s.d. - standard deviation. See Table 2 for population codes.

### *Post hoc* Environmental Association Analysis

Our final aim was to establish how local environmental effects were explicitly associated with our model of genetic structure at geographic locations. First, Pearson’s correlation analysis was performed in MINITAB ver. 14 (Minitab Inc., Pennsylvania, United States) to quantify the correlation among site-averaged climate variables (extracted from WorldClim data) - for variable pairs that were highly correlated (r≥0.75), the more biologically meaningful variable was chosen, which resulted in the selection of three uncorrelated climate variables (annual minimum temperature, annual precipitation, temperature seasonality) for *post hoc* environmental association analyses.

Next, a Multiple Regression Model (MRM) approach was used in the program GESTE ver. 2.0 [45], which is a Bayesian inference method based on genetic and environmental data that evaluates the effect of biotic and abiotic environmental factors on the genetic structure of populations. Specifically, GESTE estimates Fst values for each local population and relates them to environmental factors using a generalised linear model. STR data were run against the three uncorrelated population-specific environmental variables (above). Ten pilot runs of length = 5,000 were used to adjust acceptance rates for each parameter of the MCMC chain to the recommended range of 0.25–0.45. Thereafter, 10,000 iterations were performed per run, and a thinning interval of 20 was used.

Finally, redundancy analysis (RDA) was conducted with the vegan package in R to further disentangle the relative contribution of environmental and spatial components in driving genetic structure [46]–[49]. RDA is an analogue of multivariate linear regression analyses (above), utilising matrices of dependent and independent (explanatory) variables. The dependent matrix is represented by the genotypic data, and the explanatory variables are a spatial (*X* and *Y* coordinates) or environmental matrix. Genetic data and population-specific climate data (the three uncorrelated climate variables, annual minimum temperature, annual precipitation, and temperature seasonality) were compared in a partial RDA, specifying geography (latitude and longitude) as a third ‘conditioned’ matrix so that the analysis conditions on geographic location. To examine how much of the genetic variation in *P. pacificus* is uniquely explained by climate, how much is uniquely explained by geography, and how much is due to the combined effect of the two, we then partitioned the variance components of our RDA by running 3 models: a full model with all important climate and geographic variables as explanatory variables; a partial model in which geography explains genetic data conditioned on important climate variables; a partial model in which important climate variables explain genetic data conditioned on geography [50]. This analysis allowed us to distinguish between how much of the total explainable genetic variance was due to climate (after removing geological effects), how much was due to geography (after removing climate effects) and how much was due to the joint effect of both factors.

## Results

### Species Distribution Models (SDMs)

Continuous predictions of the potential distribution of *P. pacificus* on La Réunion Island were generated in DIVA-GIS, based on presence data at 23 geographic locations and three combinations of climatic variables (see Methods; Fig. 2a; Table 1). These were fairly consistent regardless of the combination of climate variables, hence, only the analysis using all bioclimatic variables is shown here (Fig. 2a). Collectively, the SDMs indicated that a significant proportion of the Réunion landscape has a presence probability for *P. pacificus* of ‘excellent’ or ‘very high’ (red or orange colours in Fig. 2a, corresponding to the upper two of five levels in a linear series, where level 1 represents the minimum probability value of *P. pacificus* presence and level 5, the maximum probability presence value, according to a ‘percentile envelope’ based on the various climate variables in the model). Note that in Fig. 2a, the legend indicates these presence probabilities by colour. For example, category 1 = low (0–2.5 percentile) indicates a low presence probability of *P. pacificus*, where dark green areas on the map are those corresponding to climate envelope outliers (in the <2.5 and >97.5 percentile). The areas of highest probabilities of occurrence largely corresponded to discrete pockets of habitat across an inner circular belt of the island, where conditions are more generally cooler and wetter (Fig. 2b). Conversely, the greater proportion of low-lying coastal areas, where conditions are hotter and drier (i.e. maximum temperature of warmest month >25°C, precipitation of driest quarter <150 mm), appear to be largely avoided by *P. pacificus* and/or its beetle hosts (Fig. 2b), and indeed, were deemed as not suitable or low probability areas for *P. pacificus* and beetle occurrence (Fig. 2).

**Figure 3:**
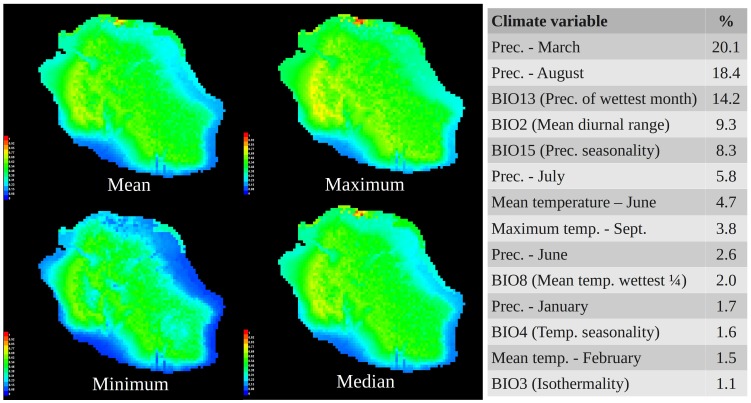
SDMs of *Pristionchus pacificus* using MAXENT. Geographic distribution of theoretical suitable habitat of *P. pacificus* on La Réunion Island, draped over a topographical surface at 2.5 arc minute resolution using the program MAXENT. In each figure, red colours indicate a high probability of suitable conditions for the species, green indicates conditions typical of those where the species is found, and lighter shades of blue indicate low predicted probability of suitable conditions. Presented are the mean, maximum, minimum and median maps generated from ten replicate runs; the table to the right shows the most important (>1%) contributing variables over these ten predications, and their percentage contribution to the model.

MAXENT predictions agreed well with the model produced by DIVA-GIS (Fig. 3), again identifying a circular band of most ideal habitat on La Réunion Island, and emphasising the south-west as particularly suitable habitat (yellow-orange colours in Fig. 3, corresponding to areas with a higher probability of suitable conditions for *P. pacificus* cf. green and blue areas). AUC values exceeded 0.70 in all analyses, indicating that MAXENT models had acceptable predictive power. Throughout the predictions, the highest contributing climate variables were always related to precipitation, regardless of the combination of variables used in the model. In the presented model (where all climate variables were employed) precipitation-based variables contributed 71.1% to the model (Fig. 3). Taken together, the SDM analyses indicated that environmental factors clearly govern the presence of *P. pacificus* in Réunion habitats, with precipitation being a particularly important predictor of species presence in different areas of the island.

### Population Genetic Structure

**Figure 4:**
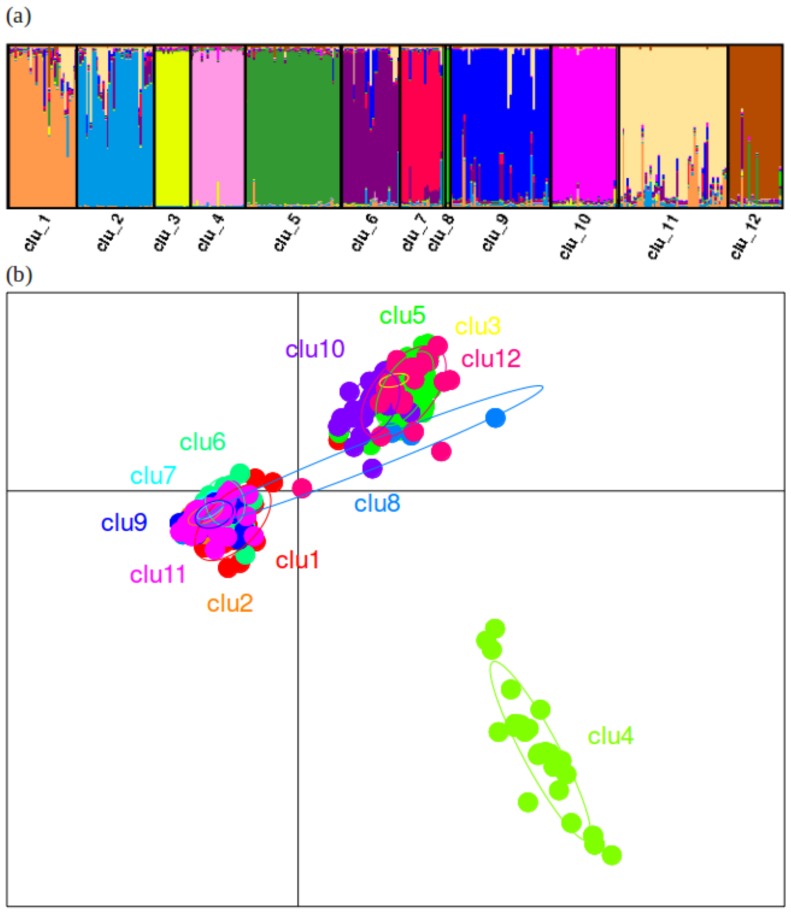
Overall population structure of *Pristionchus pacificus*. (a) Clustering analyses performed using INSTRUCT to determine the optimal number of genetic clusters in the *P. pacificus* meta-population on La Réunion Island. In the graphic, each vertical bar represents a *P. pacificus* strain and the coloured segments of each bar represent the proportion of that strains’ genetic membership to a particular cluster (n = 12; ‘clu’ = cluster); (b) Principal Component Analysis (PCA), performed using the adegenet package in R to seek a summary of the diversity among *P. pacificus* genetic clusters – axes 1 and 2 did not show clear differentiation among populations, hence PCAs of axes 1 and 3 are shown here. Note that the clusters across panels (a) and (b) are not equivalent (i.e. cluster 1 in (a), derived in InStruct ≠ cluster 1 in (b), derived from PCA).

Next, we wished to examine the effects of environmental factors on genetic structure in *P. pacificus*. INSTRUCT analyses on the non-partitioned dataset resulted in an optimal number of STR genetic clusters among *P. pacificus* La Réunion populations of 12 (Fig. 4a; Table 3). The same number of clusters was also identified in PCA analysis (Fig. 4b), but note that the results presented in Fig. 4b (PCA) do not correspond with those in 4a (INSTRUCT). Within clusters in both methods, a substantial degree of admixture (multiple population assignments within the same cluster in Figure 4a; overlapping ellipses in Figure 4b) was observed; indicating that population structure in *P. pacificus* is complex. This was evident in the PCA, where the first few axes explained only a very small proportion of the overall genetic variance (4.41, 3.67, and 3.34% for axes 1, 2 and 3, respectively) (Fig. 4b). Measures of diversity were high, with expected heterozygosity among clusters ranging from 0.149–0.635 (Table 4). Differentiation among clusters was also very large and significant (average Rst values: 0.497–0.778), supporting previous findings of low gene flow [25] (Table 5).

**Table 5:** Pair-wise Rst.

(a)	**Pop**	*eco1*	*eco2*	*eco3*	*eco4*	*eco5*	*eco6*												
	**eco1**	0.508																	
	**eco2**	*0.507*	0.412																
	**eco3**	*0.452*	0.020	0.472															
	**eco4**	0.199	*0.613*	*0.602*	0.461														
	**eco5**	*0.565*	*0.384*	*0.337*	*0.897*	0.628													
	**eco6**	0.018	*0.534*	*0.501*	*0.465*	*0.835*	0.584												
	**Pop**	*BV*	CC	CK	CO	ES	FPI	GE	NB	PA0	PA1	PC	PL	SB	SS1	SS2	TB	TBG	TK
(b)	**BV**	0.465																	
	**CC**	*0.328*	0.576																
	**CK**	*0.263*	0.958	0.497															
	**CO**	*0.521*	*0.596*	*0.540*	0.520														
	**ES**	*0.597*	*0.931*	*0.762*	0.042	0.652													
	**FPI**	*0.677*	*0.969*	*0.864*	0.072	*0.435*	0.734												
	**GE**	0.008	*0.163*	*0.206*	*0.468*	*0.528*	*0.590*	0.394											
	**NB**	*0.556*	*0.211*	0.253	*0.644*	*0.842*	*0.897*	*0.420*	0.618										
	**PA0**	0.079	*0.614*	0.369	*0.536*	0.713	*0.848*	0.020	*0.678*	0.575									
	**PA1**	*0.439*	*0.777*	*0.585*	0.061	0.322	*0.604*	*0.314*	*0.728*	*0.587*	0.551								
	**PC**	*0.564*	*0.735*	*0.661*	0.032	0.097	0.249	*0.429*	*0.755*	*0.642*	0.081	0.593							
	**PL**	0.022	*0.546*	*0.431*	*0.635*	*0.802*	*0.859*	0.040	*0.671*	0.057	*0.674*	*0.715*	0.667						
	**SB**	0.005	*0.167*	*0.223*	*0.466*	*0.507*	*0.549*	0.037	*0.448*	0.037	*0.329*	*0.441*	0.071	0.400					
	**SS1**	*0.369*	*0.366*	*0.319*	*0.246*	*0.460*	*0.619*	*0.265*	*0.510*	*0.463*	0.108	*0.322*	*0.569*	*0.302*	0.393				
	**SS2**	*0.642*	*0.901*	*0.765*	0.007	0.032	0.270	*0.554*	*0.832*	*0.749*	*0.393*	0.181	*0.808*	*0.534*	*0.503*	0.679			
	**TB**	*0.505*	*0.532*	*0.523*	0.004	0.031	0.176	*0.432*	*0.617*	*0.521*	0.024	0.008	*0.610*	*0.459*	*0.206*	0.110	0.494		
	**TBG**	*0.510*	*0.714*	*0.634*	0.076	0.147	0.337	*0.354*	*0.738*	*0.612*	0.048	0.012	*0.682*	*0.372*	*0.282*	0.200	0.027	0.553	
	**TK**	0.077	*0.665*	*0.422*	*0.551*	0.774	*0.891*	0.051	*0.699*	0.096	*0.633*	*0.661*	0.038	0.007	*0.492*	*0.784*	*0.533*	*0.632*	0.633
(c)	**Pop**	*alt1*	*alt2*	*alt3*	*alt4*	*alt5*													
	**alt1**	0.356																	
	**alt2**	0.035	0.227																
	**alt3**	*0.331*	*0.194*	0.405															
	**alt4**	*0.391*	*0.233*	0.006	0.401														
	**alt5**	*0.347*	*0.254*	*0.689*	*0.579*	0.467													
(d)	**Pop**	*ado*	*amn*	*aph*	*ata*	*hch*	*hop*	*mal*	*ory*	*soi*									
	**ado**	0.232																	
	**amn**	*0.261*	0.538																
	**aph**	*0.261*	*0.643*	0.456															
	**ata**	*0.270*	*0.687*	0.003	0.487														
	**hch**	0.055	*0.487*	*0.512*	*0.544*	0.500													
	**hop**	*0.130*	0.139	*0.577*	*0.619*	0.163	0.478												
	**mal**	*0.236*	*0.525*	0.013	0.009	*0.457*	*0.474*	0.393											
	**ory**	0.041	*0.433*	*0.286*	*0.316*	0.063	0.190	*0.276*	0.328										
	**soi**	0.196	*0.733*	0.258	0.186	0.155	*0.589*	0.183	0.359	0.661									
(e)	**Pop**	*clu1*	*clu2*	*clu3*	*clu4*	*clu5*	*clu6*	*clu7*	*clu8*	*clu9*	*clu10*	*clu11*	*clu12*						
	**clu1**	0.529																	
	**clu2**	*0.150*	0.527																
	**clu3**	*0.588*	*0.660*	0.731															
	**clu4**	*0.760*	*0.860*	*0.933*	0.761														
	**clu5**	*0.773*	*0.835*	*0.805*	*0.482*	0.778													
	**clu6**	*0.153*	*0.512*	*0.817*	*0.904*	*0.906*	0.630												
	**clu7**	0.067	*0.404*	*0.852*	*0.929*	*0.919*	0.097	0.645											
	**clu8**	*0.496*	*0.629*	*0.711*	*0.788*	*0.611*	*0.751*	*0.760*	0.667										
	**clu9**	0.004	*0.136*	*0.728*	*0.864*	*0.851*	*0.343*	*0.196*	*0.674*	0.540									
	**clu10**	*0.632*	*0.641*	0.153	*0.832*	*0.802*	*0.800*	*0.899*	*0.668*	*0.724*	0.725								
	**clu11**	0.059	*0.241*	*0.663*	*0.803*	*0.795*	*0.268*	*0.100*	*0.586*	*0.108*	*0.677*	0.497							
	**clu12**	*0.677*	*0.734*	*0.548*	*0.216*	0.038	*0.844*	*0.847*	0.315	*0.775*	*0.675*	*0.725*	0.671						

Pair-wise Rst values among *Pristionchus pacificus* populations for STR datasets: (a) ecozone; (b) geography; (c) altitude; (d) beetle host; and (e) genetic cluster. Significant values are shown in *italics*; underlined diagonal is average significant differentiation of each population to all other populations (see Table 2 for population codes).

**Figure 5:**
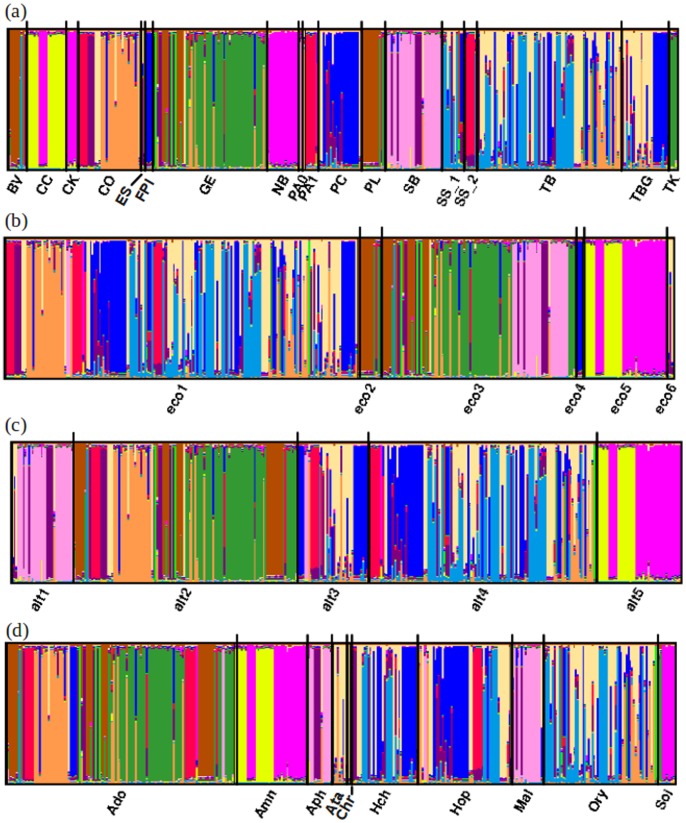
Clustering analyses by environment for *Pristionchus pacificus*. Results from INSTRUCT re-coloured according to genetic cluster (see Fig. 3a) to determine the relationship between cluster and environmental factors in *P. pacificus* on La Réunion Island. In the graphic, each vertical bar represents a *P. pacificus* strain and the coloured segments of each bar represent the proportion of that strains’ genetic membership to a particular cluster. Results are presented based on: (a) geography (see Fig. 1 and Table 2 for location codes); (b) ecozone (‘eco’ = ecozone); (c) altitude (‘alt’ = altitude); and (d) beetle host (see Table 2 for beetle codes).

**Figure 6:**
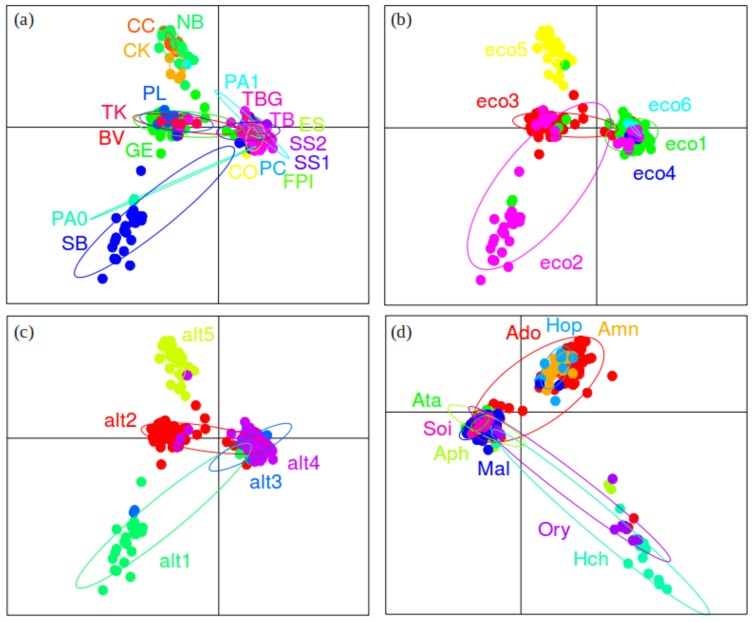
PCA analyses by environment for *Pristionchus pacificus*. Principal Component Analysis (PCA) performed using the adegenet package in R to seek a summary of the genetic diversity, and its relationship to environmental factors, in *P. pacificus* on La Réunion Island. Results represented based on: (a) geography (see Fig. 1 and Table 2 for location codes); (b) ecozone (‘eco’ = ecozone); (c) altitude (‘alt’ = altitude); and (d) beetle host (see Table 2 for beetle codes). In each case, axes 1 and 2 did not show clear differentiation among populations, hence PCAs of axes 1 and 3 are shown.

When INSTRUCT results were reproduced according to environmentally-relevant definitions of population structure, we found that environmental factors influence genetic structure in *P. pacificus* (Fig. 5,6). For example, the colours in Figure 5a show several instances of geographically local clustering at locations GE (green), SB (light pink/purple), and TB (light blue). Ecozone is also responsible for a significant proportion of genetic structure in *P. pacificus*; only brown, dark blue and dark pink colours are found across ecozone divisions in Figure 5b. Structuring by altitude and beetle re-iterated this pattern, where a broad amount of diversity was present within each altitude or beetle group, but less diversity was shared across each classification (Fig. 5c,d). These results were confirmed in PCA analyses, where broad differentiation patterns among geographic-, ecozone-, altitude- and beetle-defined populations were also found (Fig. 6). In particular, four distinct genetic clusters defined by geographic location were present: {SB/PA0}, {BV/GE/PL/TK}, {CC/CK/NB}, {PC/TB/TBG/SS1/SS2/PA1/ES/FPI} (Fig. 6a). Four ecozone groups {2}, {3}, {5} and {1/4/6} showed differing axes of differentiation (Fig. 6b), as did four altitude groups {1}, {2}, {3/4}, and {5}; Fig. 6c). The final PCA also separated several broad groups of genetic structure partitioned among beetle host: {Ado/Hop/Amn}, {Ory/Hch}, {Ata/Aph/Mal/Soi}; Fig. 6d). However, as for the PCA on the non-partitioned dataset (Fig. 4b), the overall variance explained by the first few principal components in these subsequent PCA analyses was low (cumulative inertia for axes 1∶3 of 11.42–11.63% for all datasets) (Fig. 6). When PCA analyses were re-run for reduced cluster, ecozone, and geographic location datasets (removing all populations for which n<5; see Methods), no significant differences were detected, indicating that this analysis was robust to unbalanced sample sizes (data not shown). Analyses in ARLEQUIN further supported the INSTRUCT/PCA results, as all datasets (ecozone, geography, altitude and beetle) produced findings of high diversity within populations, and strong significant differentiation (Rst >0.400) between them (Table 4,5). In AMOVAs, all datasets partitioned large (27.23–45.33%), significant (*P*<0.001) amounts of genetic variation (Table 6), indicating that ecozone, altitude, geography and beetle are all important forces in genetic structuring among La Réunion *P. pacificus*.

**Table 6:** Analysis of molecular variance.

Population definition	Df	SS	Var	%
Beetle	8	52,696	81.23	27.23
Altitude	4	48,465	87.22	28.39
Geographic location	17	83,944	118.33	39.76
Ecozone	5	75,787	152.15	45.33

ANOVA results for the partitioning of genetic diversity among *Pristionchus pacificus* La Réunion populations over four separate tests, where populations are defined by beetle, altitude, geographic location or ecozone.Df – degrees of freedom; SS – sum of squares; Var – variance component among populations; % - percentage of variation among populations (NB: the percentage of variation within populations = 1-%); all results are statistically significant (*P*<0.001).

Although environmental factors clearly contribute to the genetic structure in *P. pacificus*, the INSTRUCT and PCA results also demonstrated that genetic diversity among ‘populations’ has been strongly impacted by the historical mixing of lineages. This is shown, for example, by the lack of an overall clear relationship between genetic cluster and geographic location – most clusters are spread over at least four geographic locations (mean = 4.7), and one cluster was found in nine locations (Fig. 5a; Table 2). In PCAs, the geographic dataset differentiated four groups (above), but ellipses generally showed a large degree of overlap between these groups (Fig. 6a). Such complex patterns were found for all the datasets. For example, ecozones 2 (PL, SB), 3 (BV, GE, TK) and 5 (CC, CK, NB) were differentiated from, but often overlapped with ecozones 1/4/6 (Fig. 5, 6). The pattern for the geographic dataset corresponded to lineages A and C co-existing at SB/PA0, lineage B forming its own group (CC, CK and NB), combinations of lineages A, C and D co-occurring at (BV/GE/PL/TK), and the remaining group containing either lineage C or lineage C and D individuals (Fig. 1c; Table 2). Thus, the sharing of historical (lineage) diversity across geographic locations, altitudes, ecozones and beetle hosts (Fig. 5, 6) complicates potential patterning of geographic and environmental population genetic structure.

### *Post hoc* Environmental Association Analysis

Next, we wished to see whether local environmental effects were associated with genetic structure despite constraints associated with the historical diversification of lineages. Regression approaches employed in GESTE found that runs with just a single environmental factor did not produce higher probability models when the factor was included in the model compared to when it was excluded (Table 7). However, two-factor tests found the highest probability models to be those that included both factors and their interaction term in all cases. When the factors were annual minimum temperature and annual precipitation, their interaction explained the highest proportion of genetic variation in the model, while the individual factors contributed more than their interactions when annual minimum temperature or annual precipitation were considered alongside temperature seasonality (Table 7). Finally, the three-factor test identified the highest probability model as one including precipitation, where all three variables explained similar amounts of genetic variation (Table 7).

**Table 7:** GESTE results.

Factor(s) in model (G)	Highest probability model	P(model)	P(G1)	P(G2)	P(G1*G2)	P(G3)
Tmin	Constant	0.522	0.478			
Prec	Constant	0.509	0.491			
Temp_s	Constant	0.524	0.476			
Tmin, Prec	Constant, G1, G2, G1*G2	0.478	0.251	0.260	0.478	
Tmin, Temp_s	Constant, G1, G2, G1*G2	0.258	0.361	0.359	0.258	
Prec, Temp_s	Constant, G1, G2, G1*G2	0.270	0.354	0.364	0.270	
Tmin, Prec, Temp_s	Constant, G2	0.132	0.499	0.498	–	0.495

Results of seven regression analyses performed in GESTE to examine how much of the variation in genetic structure among *Pristionchus pacificus* populations on La Réunion Island is explained by variation in environmental factors. The first column shows the environmental factor(s) tested in a given analysis. The second and third columns correspond to the model which best explains the data (where the constant term corresponds to bias that is not accounted for by the terms in the model), and its probability (P), respectively. The remaining four columns give the probability of each individual factor in the highest probability model, with the factor in the far left column corresponding (from left to right) with labels G1, G2 and/or G3 in subsequent columns, and G1*G2 referring to the interaction between factors G1 and G2. Thus, in the first three tests, the probability of the individual factors (environmental variables) in the model is less than that of the a model including only random (constant) effects, whereas in the subsequent two-factor tests, the highest probability models were those that included both factors and their interaction (see Results for further information).

**Figure 7:**
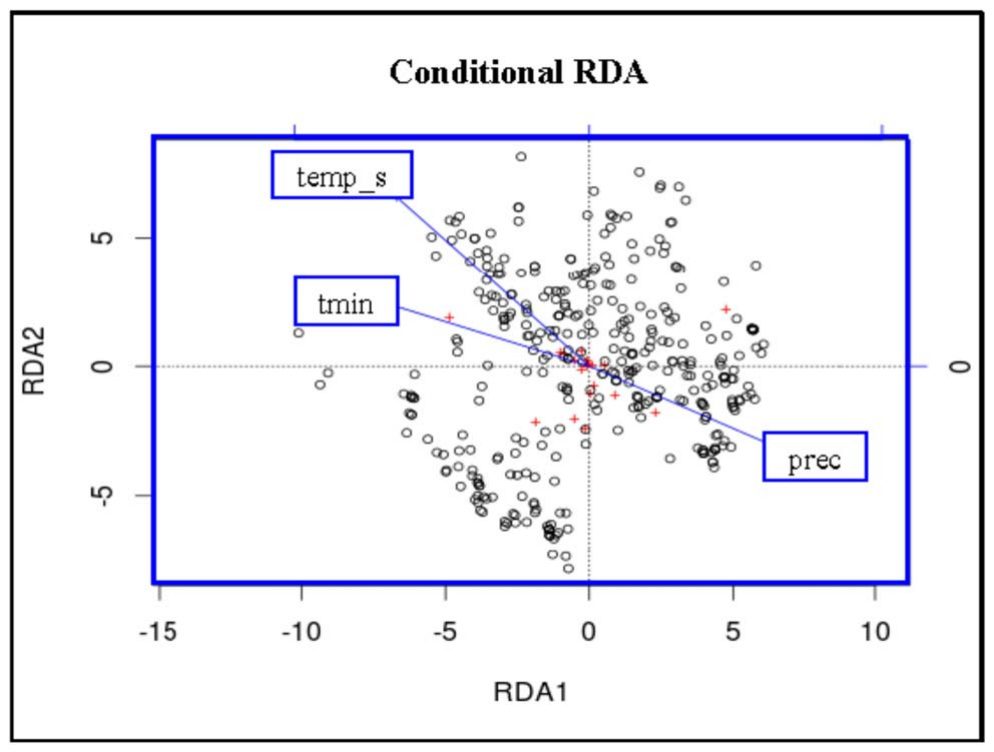
RDA analyses for *Pristionchus pacificus*. Redundancy analysis (RDA) performed using the vegan package in R to determine the relative contribution of environmental and spatial components driving genetic structure in *P. pacificus*. The biplot depicts the eigenvalues and lengths of eigenvectors for the RDA conditioned on geography (latitude and longitude); tmin = annual minimum temperature, prec = annual precipitation, temp_s = temperature seasonality. See Results for more information.

Partial RDA analysis conditioned on geography (latitude/longitude), found a significant association between climate and STR genotype (F_3,361_ = 13.91; *P = *0.005; Fig. 7). Partitioning of total variance analysis (comparing the full model with a partial model conditioned on climate and a partial model conditioned on geography) indicated that climate explains 65.4% of the total explainable genetic variance after removing variance explained by geography; geography explains 32.0% of total variance after removing variance explained by climate; and climate and geography together have a joint effect of 2.6% on genetic variance (this is also the proportion of variance in which climate and geography cannot be separated due to collinearity).

## Discussion

Combined analysis of genetic data, multivariate statistics using environmental data, and species distribution modelling, suggests that current genetic variation patterns in *P. pacificus* from La Réunion Island may be strongly influenced by local environmental conditions. In particular, environmental components appear to have a stronger effect than geographic distance-based isolating factors such as genetic drift.

SDMs highlighted environmental distinctiveness and habitat discontinuities among *P. pacificus* putative habitats on Réunion Island. The discrete nature of suitable conditions corresponded to an inner circular belt of the island where the potential niche of *P. pacificus* would be generally cool and wet. Active avoidance of hotter, drier areas by both *P. pacificus* and its beetle hosts supported the earlier observation of demarcation between western (drier) and eastern (wetter) zones of the island [26]. However, the distribution models showed that, rather than a simple divide bisecting eastern and western regions, suitable *P. pacificus* habitat is partitioned across local ecozones. This was also supported by our AMOVA results, for which the dataset defined by ecozone explained approximately 45% of regional genetic variation, some 20% higher than altitude or beetle-defined datasets, and ∼6% higher than the variation explained by geographic location. The question of whether the environmental preferences borne out from our SDM analysis reflect those of the nematode or its beetle hosts is difficult to address because beetle host, ecozone and altitude cannot easily be dissociated. Therefore, host affinity among different *P. pacificus* genotypes may provide a stronger pressure than the environment *per* se. Recent work has tested this idea using chemoattraction assays to determine whether nematodes might be preferentially attracted towards beetle washes from their original host beetle species [51]. While this work found that strains respond similarly to each other when presented with beetle washes than with off-the-shelf organic compounds, no clear signal for original host preference was detected [51]. The AMOVA results reported here support this in that beetle host species accounts for the lowest percentage (27.23%) of regional genetic variation compared to the other environmentally-defined datasets.

Our analysis recovered 12 genetic clusters from the La Réunion *P. pacificus* meta-population. These distinct clusters were themselves characterised by high genetic diversity, supporting previous results at both mitochondrial and STR markers [23], [26]. When populations were defined by various environmental classification schemes, diversity remained high, suggesting a strong role for the environment in the mediation and maintenance of genetic structure. However, this relationship was complex, as single genetic clusters generally contributed diversity to multiple populations. This was particularly evident for analyses based on geographic location, where up to nine genetic clusters contributed to a single population. INSTRUCT and PCA results both demonstrated that genetic diversity among ecozones, altitudes and beetle hosts has also been strongly impacted by the historical mixing of lineages. In particular, the mixed genetic signal meant that only a small degree of genetic variance could be extracted for each PCA axis, regardless of how population was defined. The co-existence of genetic lineages across habitats complicates potential patterning of geographic and environmental population structure. However, despite historical genetic mixing, current differentiation among clusters, geographic locations, ecozones, altitudes and beetle hosts was very large and significant, supporting previous findings of low gene flow [26]. Future work based on further sample collections should examine the potential relationship between genetic lineage and environmental factors to further tease apart the independent roles of evolutionary heritage, demography and environment in partitioning genetic structure.

Given the strong genetic diversity within locations, it is surprising that we found signals of significant association between environmental and genetic factors at each location. This was first hinted at in the SDMs performed with MAXENT software, which, in addition to helping characterise the environmental niche of *P. pacificus*, suggested that precipitation-based variables were very important in determining suitable habitat for *P. pacificus*. To quantify the importance of environmental factors to the genetic structure of populations, we employed various *post hoc* association tests. These demonstrated the presence of strong correlations between environmental and genetic distances among geographic populations and suggested that patterns beyond drift in isolated populations are important determinants of genetic structure. In fact, climate was shown in redundancy analysis to explain ∼65% of the total explainable genetic variance after removing variance explained by geography.

Significant correlations between genetic and environmental variation may arise when habitat differentiation acts as a barrier to gene flow, causing environmental isolation and genetic differentiation even when populations are spatially close [5]. Although association does not imply causality, the primary signature of adaptive evolution through time is the deterministic change in allele frequencies among populations. Thus, the observed association of genetic differentiation with environmental gradients may suggest a role for natural selection in shaping the divergence of *P. pacificus* populations. In fact, association between putatively neutral genetic variation and environmental variation can be interpreted as evidence of diversifying selection acting over the whole genome [5]. Further, a significant positive partial correlation after removing the effect of geographic distance suggests that genetic divergence is associated with environmental gradients and that natural selection may interact with neutral processes of gene flow and genetic drift [52]–[56]. Indeed, recent work has highlighted the importance of non-neutral, deleterious processes operating in the *P. pacificus* genome [57]. A highly selfing lifestyle, coupled with the influence of periodic out-crossing in creating new genetic assemblages [26], is thought to have increased the adaptive potential of *P. pacificus*, which appears to be highly diverse genetically compared to other nematode taxa (e.g. *Caenorhabditis elegans* [58]). As such, *P. pacificus*, with its wide pool of raw genetic material and many opportunities for ecological and environmental specialisation, seems a prime target for adaptive divergence in a setting such as La Réunion Island.

Environmental heterogeneity facilitating adaptive divergence represents an important component of natural populations today, especially in the face of proposed climate changes. By imposing diverse selection pressures across geographic regions, environmental factors can affect genetic structure and distribution as shown here and in [5], [9], but also host-pathogen interactions [6], gene flow and local adaption [7], [8], [59]–[61]. As such, the environment represents a key role that should be increasingly incorporated into evolutionary studies. Indeed, ‘landscape genetics’ is an emerging discipline that examines quantitative effects of landscape or climatic variables on gene flow among populations or individuals [7], [12]–[16]. Such studies represent a powerful approach for understanding the environment as a proximate cause of evolutionary processes.

In summary, we aimed to explicitly test the hypothesis that genetic structure in *P. pacificus* is influenced by environmental variables. Working with a large genome-spanning STR dataset and covering a broad geographic range of populations enabled us to evaluate the effects of environmental factors on genetic structure. Specifically, we saw how interactions between genetic diversity, multiple introductions, reproductive mode and isolation by environment are together involved in the response of *P. pacificus* to local environments. Environmental correlations with neutral genetic markers can help identify opportunities for natural selection (e.g. [59]), but the mapping of adaptive genetic variation that underlies the phenotypes under selection provides even stronger evidence [e.g. 60–61]. Future work with *P. pacificus* in the La Réunion Island setting has the potential to link genomic variation with functional studies to explore the adaptive processes and mechanisms driving present-day population genomic structure in more detail.
